# Oncogenic effects of ECM remodeling in obesity and breast cancer

**DOI:** 10.1038/s41388-025-03521-x

**Published:** 2025-08-22

**Authors:** Mackenzie L. Hawes, Marcus A. Moody, Caroline R. McCauley, Abigail G. Huddleston, Maansi Solanky, Dara H. Khosravi, Ayushi R. Patel, Ronald M. Lynch, Suresh K. Alahari, Bruce A. Bunnell, Jorge A. Belgodere, Van T. Hoang, Matthew E. Burow, Elizabeth C. Martin

**Affiliations:** 1 Department of Medicine, Section of Hematology and Oncology, Tulane University School of Medicine, New Orleans, LA USA; 2 Department of Microbiology, Immunology and Genetics, University of North Texas Health Science Center, Fort Worth, TX USA; 3 School of Medicine, LSU Health Science Center, New Orleans, LA USA; 4 Cardiovascular Center of Excellence, Louisiana State University Health Sciences Center, New Orleans, LA USA; 5 Department of Pharmacology and Experimental Therapeutics, Louisiana State University Health Sciences Center, New Orleans, LA USA; 6 Departments of Physiology and Pharmacology, at the University of Arizona Cancer Center, University of Arizona, Tucson, AZ USA; 7 Department of Biochemistry and Molecular Biology, LSU Health Science Center School of Medicine, New Orleans, LA USA; 8 Stanley S. Scott Cancer Center, LSU Health Science Center School of Medicine, New Orleans, LA USA; 9 Tulane Cancer Center, Tulane University, New Orleans, LA USA; 10 Department of Biological and Agricultural Engineering, Louisiana State University and Agricultural Center, Baton Rouge, LA USA

**Keywords:** Breast cancer, Tumour heterogeneity, Cancer models, Cancer microenvironment

## Abstract

Extracellular matrix (ECM) components are key regulators in breast cancer progression, as ECM remodeling is essential for breast cancer cells to invade into surrounding tissue. This process is characterized by the alignment of fibrillar collagens, breakdown of basement membrane components, and increased interstitial collagen stiffness. In patients with obesity, pre-existing ECM changes, including excessive collagen deposition and heightened matrix stiffness, mimic alterations detected in breast cancer. Given that obesity is a predictor of poor prognosis and resistance to treatment in breast cancer, it is crucial to understand how ECM conditioned by obesity affects disease outcomes. In this review, we highlight known ECM changes that occur with breast cancer and obesity and describe how these changes impact cancer cell metastasis, disease progression, and the breast cancer tumor microenvironment. We examine how obesity driven ECM remodeling affects treatment response and resistance. Further, we discuss how the compounding factor of age contributes to remodeling and current preclinical models of ECM in breast cancer.

## Introduction

As of 2020, 42% of the global population is classified as obese (BMI > 30), a statistic projected to grow to 54% by 2035 (World Obesity Atlas). In all breast cancer subtypes, obesity is an indicator of poor overall survival; however, the risk of developing breast cancer is heightened only in post-menopausal women with obesity. Pre-menopausal women with obesity are less likely than post-menopausal women with obesity to develop breast cancer, but they retain poor disease outcomes after diagnosis [1]. Therefore, obesity is a prognostic marker for breast cancer, and with the proportion of people with obesity, it is essential to understand how this condition alters the underlying structure and function of the breast.

The breast tumor microenvironment (TME) is composed of adipose tissue, stromal cells, immune cells, and extracellular matrix (ECM) [2]. In the obese, breast TME, strain from enlarging adipocytes on the ECM induces local hypoxia and inflammation, prompting the release of excessive ECM proteins [3, 4]. Dysregulation of ECM deposition from obese stromal cells results in randomly aligned, thickened, and highly crosslinked collagen fibers that increase stiffness in the interstitial matrix [3, 5, 6]. At the tumor-stroma interface, ECM remodeling by breast cancer cells is stage-dependent. Early-stage tumors induce fibrosis, increasing matrix stiffness from less than 100 Pa to over 4 kPa [7–9]. Late-stage breast tumors remain stiff and initiate invasion through local alignment of collagen fibers and breakdown of basement membrane components [9–11]. Combined, obesity and breast cancer promote increased breast density and stiffness, with local and stage-dependent changes to ECM proteins. Cancer cells can then act upon this remodeled matrix through mechano-sensors, including integrins and focal adhesions, which recognize structural changes in the matrix and initiate downstream signaling in cancer cells [12].

This review highlights how compositional changes, distinct structure and alignment, and mechanical features of the obese ECM alter breast cancer progression. We further explore the role of non-cancer stromal cell types in facilitating ECM remodeling during obesity and the impact of altered ECM on breast cancer treatment efficacy. As menopause status dictates whether obesity is a risk factor for breast cancer development, we discuss age-induced changes to the ECM and adipose tissue. Finally, we provide insights on new and existing techniques for analyzing the ECM in the context of obesity and breast cancer.

## Compositional features of the obese ECM

**Fig. 1:**
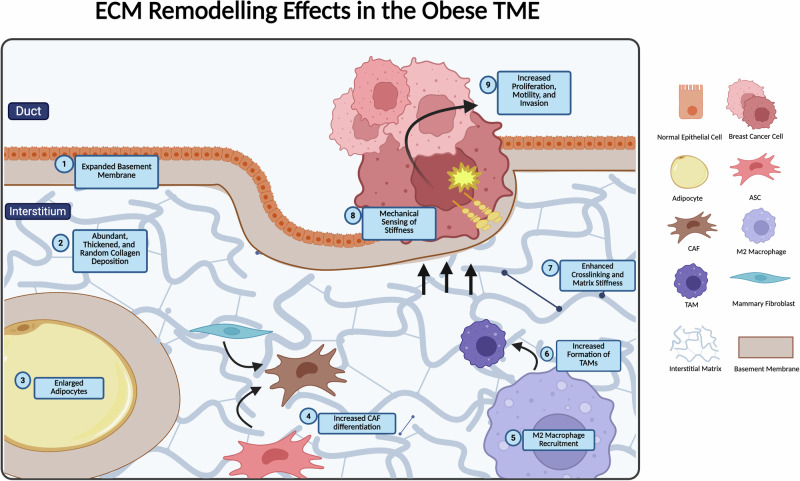
ECM remodeling effects in the obese TME. The obese TME is marked by a variety of changes to the ECM architecture and stromal components. Obesity induces alterations to the ECM that not only affect the structure of the tumor-stroma but also drive recruitment of new cell types, which further modify the TME. The cumulative effect of recruitment and remodeling leads to a TME that is fundamentally distinct from that of lean individuals. Structural changes to the TME include expansion and excessive deposition of basement membrane components (see 1), a highly fibrotic matrix that is characterized by thickened and randomly aligned collagen fibers (see 2), enhanced collagen crosslinking by lysyl oxidases, and an increase in total matrix stiffness (see 7). Obesity-induced changes to the local cell populations include an increase in adipocyte size (see 3), a heightened predisposition for CAF differentiation from ASCs (see 4), and recruitment of M2 macrophage subtypes to the TME and subsequent TAM formation (see 5 and 6). The resulting pressure from increased size and ECM deposition from these cell types further contributes to ECM stiffening, an impact that is recognized by force-sensing machinery within the cancer cell and promotes pro-tumorigenic signaling and eventual disease progression (see 8 and 9).

Obesity is marked by dynamic changes to adipose tissue, including expansion of energy-storing white adipose tissue (WAT) and the dysregulation of temperature-regulating brown adipose tissue (BAT) [13]. Adipose tissues contain a heterogeneous population of cells embedded within a protein-rich ECM with various collagens, laminins, and fibronectins. The surrounding environment provides biochemical and biomechanical cues that nurture cell and tissue growth through balanced homeostatic processes [14, 15]. As the adipose tissue expands in size and density, the existing extracellular matrix is remodeled to accommodate larger adipocytes and increased tissue mass (Fig. 1) [16].

ECM remodeling by obesity leads to the accumulation of fibrotic components that promote metabolic dysregulation within adipose tissue. Collagens make up 50–90% of the extracellular matrix and are abnormally deposited in the obese adipose tissue [16, 17]. RNA-sequencing of human subcutaneous white adipose tissue revealed that obesity increases expression of collagen genes COL4A2, COL5A1, COL5A2, COL6A1, COL12A1, and COL16A1. The unique upregulation of COL1A2, COL4A1, COL5A3, COL6A2, and COL15 in obese visceral white adipose tissue further highlights the depot-specific nature of obesity-associated ECM remodeling [16]. Increases in COL4A1, a prominent basement membrane scaffolding protein in adipocytes, are more abundant in obese subcutaneous versus visceral-derived adipocytes. Interestingly, these changes are associated with markers of glucose homeostasis and insulin resistance in subcutaneous depots [18].

The basement membrane of obese subcutaneous adipocytes is more densely populated than in lean and exhibits upregulated basement membrane gene expression, including secreted protein rich in cysteine (SPARC), laminin subunit gamma 1 (LAMC1), nidogen-1 (NID-1), and heparin sulfate proteoglycan-2 (HSPG-2) [18]. Laminin isoform alpha 4 (LAMA4) expression, a key component of the basement membrane, is higher in patients with obesity and in vivo obesity models [19, 20]. Interestingly, weight loss in obese mice and humans does not decrease LAMA4 expression, indicating potential lasting effects of obesity [19]. Moreover, LAMA4 knockout results in the inability of mice fed a high-fat diet (HFD) to gain weight. These mice have less epididymal fat present overall, indicating the importance of laminins and other ECM proteins in developing and maintaining obesity [21].

Studies analyzing obesity and ECM remodeling most often examine abdominally derived adipose tissue and adipocytes. While this tissue analysis provides insight into the obese tissue environment, it is not an exact match for use in breast cancer studies, as the breast and abdominal adipose depots are distinct [22]. Specifically, breast-derived adipose stromal cells (ASCs) are more predisposed to osteogenic differentiation than abdominally derived ASCs [22, 23]. The obese breast matrisome in humans has not been fully characterized, but mouse mammary tissue has been thoroughly evaluated. Wishart et al. profiled decellularized mammary fat pads (MFPs) of C57BL/6 mice fed an HFD and identified 18 differentially expressed collagens with obesity. The top five genes are associated with collagens type I, III, VI, and VII (COL1A1, COL3A1, COL6A6, COL6A5, and COL7A1). Expression of glycoproteins altered by obesity in the MFP includes LAMC1, DPT, LAMA2, and NID-1, among the 27 detected [24]. Although additional studies on human breast tissue should be performed to fully elucidate ECM remodeling specifically in the obese breast, these data suggest that obesity induces elevated deposition of collagens, basement membrane expansion, and metabolic dysregulation in breast tissue (Fig. 1).

## Laboratory models for studying ECM

**Fig. 2:**
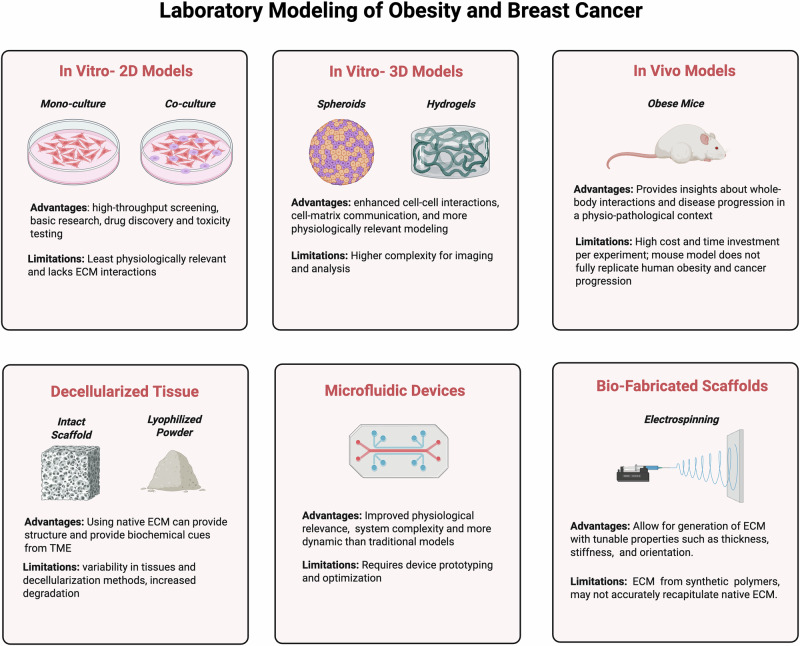
Overview of lab models of obesity and breast cancer. Several methods are used to recapitulate obesity and breast cancer within the lab. Each method has its advantages and limitations that should be considered before experimentation. In vitro methods are cost-effective and high throughput but are not physiologically relevant or accurate to the TME. 3D models enhance this relevance and allow for interactions between cells and the generation of matrix; however, the increased complexity of this system makes imaging and analysis difficult. In Vivo models of obesity, such as the ob/ob model or HFD, allow for the highest degree of relevance; however, they are expensive and have a high biological burden. Decellularization techniques allow for the use of native tissue to generate scaffolds for cell seeding. These scaffolds have a high degree of variability based on the donor tissue and the reagents/solvents used for decellularization. Micro-physiological systems offer a tunable and relevant way to study obesity and breast cancer, but often require system engineering and optimization, which can lead to variability in downstream analyses. Bio-fabricated scaffolds allow researchers to tune the ECM architecture (thickness, orientation, curvature), however, fibers generated in this process are synthetic and may not mimic native tissue ECM.

Preclinical models used to determine the oncogenic effects of ECM remodeling in obesity and breast cancer have strengths and limitations depending on the application (Fig. 2). Traditional cell culture models are high throughput, allowing investigation into large-scale analyses of cellular proliferation and drug response; however, 2D cell culture models fail to mimic the complexity of the TME, which contains several cell types and cell-cell contact in 3D [25]. Co-culture models in 2D using ASCs, fibroblasts, or immune cells can provide further insight into cell-cell interactions. These models are limited by their lack of physical and chemical cues seen in more robust 3D models. Drug response experiments may also be inaccurate in the 2D setting, as cells in 3D models differentially respond to chemotherapy treatment [25]. Models in 2D are not ideal for studies focusing on native ECM, as they require chemical induction by compounds like ascorbic acid for ECM production. The use of ECM-coated cell culture plates can be beneficial to examine the effects of a single ECM component on cancer cell biology, but they are not representative of ECM in the TME [26].

In contrast, 3D models like tumor spheroids and decellularized ECM offer insights into cell-cell interactions and ECM dynamics. Culture models in 3D produce ECM, making them more relevant for studies focusing on native ECM dynamics between breast cancer and stromal cells. Spheroids are a common 3D culture model that recapitulates tumor characteristics not observed in 2D cultures. In spheroid models, cells self-aggregate to form a tight cellular sphere, which allows for tighter cell-cell interactions. While breast cancer spheroids can replicate properties of solid tumors to some degree, hypoxia in the core limits the length of culture time to under 72 hours [27, 28]. 3D models also require more advanced imaging systems, including features such as confocal microscopy, and are more difficult to utilize in downstream biochemical analyses.

Decellularized ECM (dECM), a 3D model that preserves ECM structure and removes cellular components of both tissues and spheroids, can additionally be used to study ECM dynamics. Tissue used for this purpose can be derived from either human, animal, or cell-based sources and can be used as an intact scaffold for cell seeding and invasion assays or lyophilized into a powder [5]. While using mouse or porcine decellularized adipose is not a perfect model for human breast adipose, it is easier to obtain and a viable option [5, 29]. Lyophilized dECM can generate tissue-specific ECM in powdered form and can be utilized to generate hydrogels for use in micro-physiological systems (MPS) or as additives to 2D or 3D cell culture [28, 30]. Hydrogels with dECM can be created by solubilizing the lyophilized dECM. Embedding cells such as adipocytes in a dECM hydrogel allows the adipocytes to retain function and morphology for extended periods [30]. However, non-dECM hydrogels can be procured from other natural sources, such as Matrigel and collagen, or can be synthetically generated using polymers like PEG or PVA.

Polymeric, synthetic fibers can additionally be used to mimic the obese ECM through electrospinning. Electrospinning generates fibers with resolution at the micro- and nanometer level and can be used to make regenerated tissues. The polymer substrates are customizable, cost-effective, and can mimic the architecture of obese ECM by tuning thickness, alignment, and stiffness. This technique has mainly been used to examine invasion, migration, and proliferation in response to aligned and unaligned scaffolds, but could also offer a method of studying the architecture of the obese ECM [31].

Other methods of studying obesity, breast cancer, and ECM include MPS and microfluidic devices, which recapitulate ECM changes during tumor progression and allow for real-time monitoring [32–34]. For example, using a fibroblast-assembled ECM in a microfluidic chip induced overexpression of MMP-2 and MMP-9 in the TME, and increased fibronectin (FN1) and hyaluronan (HA) [35]. These approaches are customizable and enable the generation of models physiologically similar to human breast tissues, which can include multiple stromal cell types, either synthetic or dECM scaffolds, and imitations of the stromal vasculature, among others [30, 34].

In vivo models provide insight into cancer progression, metastasis, drug response, and ECM remodeling. While NSG/SCID mice are commonly used for breast cancer xenograft studies, variables such as the method of tumor induction and endocrine and immune response lead to differential outcomes, evidenced by primary metastasis to the lungs in mice versus to the brain and bones observed in humans [36]. Obesity models using genetically modified ob/ob or db/db mice, which lack functional leptin or leptin receptor, respectively, can replicate changes in ECM remodeling [5]. Leptin depletion should be considered when using ob/ob mice, as leptin is important in cancer-stromal crosstalk and is upregulated in obese ASCs [37]. Diet-induced obesity models, which usually consist of 45-60% fat by calories, are also appropriate to mimic obesity in vivo. However, obesity varies in this model, as the sucrose source is not standardized, differing by institution, and fatty-acid composition is not considered [38]. Additionally, observable models of native ECM in vivo, by way of surgically inserted tumor window chambers (WC), are emerging as a promising way to monitor ECM in real-time. The WC platform has been used to microscopically visualize tumor vascularization and growth in the rat breast [39]. Subsequent studies with a similar WC model used a full range of imaging modalities to quantitatively evaluate tumor growth and the TME within the mammary fat pad of mice [40, 41]. Although murine models are beneficial for studying obesity and breast cancer, they do not fully represent the human condition and disease progression. Thus, there is a need for improved in vitro models to recapitulate the TME.

## Impact of obese ECM composition on breast cancer progression

Breast cancer molecular subtypes interact with and deposit ECM in unique ways. Breast cancer is classified into four distinct molecular subtypes, based on the presence of targetable receptors within the tumor. Tumors where more than 1% of cells express the estrogen or progesterone receptor, but do not express the human epidermal growth factor 2 (HER2), are considered luminal A or B and are hormone receptor-positive (HR+). Hormone receptor-negative (HR-) breast cancers, which do not express estrogen or progesterone receptors, are considered either HER2+ or triple-negative (TNBC), depending on the presence of HER2 [42]. ECM remodeling within these subtypes is dynamic and takes part in a broader feedback loop with cancer, stromal, and immune cell signaling. The direct impacts of obesity in tumor-adjacent regions remain unexplored. Global ECM changes to the obese stroma are pro-tumorigenic and present challenges for therapy [6, 43, 44]. Independent of breast cancer, collagen types I, III, VI, and VII are markedly upregulated in obese mammary fat pads compared to lean [45]. Our prior work shows that collagen I is elevated in the HR^+^ subtypes but not enriched in TNBC tumors. In agreement with these findings, others have determined the link between collagen I and survival primarily in HR^+^ subtypes [46, 47]. Histological analysis of the breast tumor stroma and clinicopathological factors of breast cancer indicate that high collagen I expression correlates with poor overall and breast cancer-specific survival in patients treated with chemotherapy. In untreated tumors, however, collagen I expression is favorable for breast cancer-specific survival and overall survival in a cohort of predominantly HR+ breast cancer patients [48]. Collagen III knockdown in vivo promotes local invasion, alignment, and tumor growth in a murine TNBC cell line, suggesting that high expression is tumor-suppressive [49]. Recombinant collagen VII treatment on HR+ cell lines perturbs cancer cell proliferative capacity, and histological analysis indicates collagen VII may be a positive prognostic marker for breast cancer [50]. Decellularized mammary fat pads from mice fed an HFD increase the migration, cell area, and eccentricity of TNBC cells and are enriched in collagen VI [45]. Expansion of basement membrane components in obese adipose promotes expression of the matricellular protein, SPARC, and proteoglycan NID-1. The effects of SPARC on breast cancer are contested, as SPARC has been associated with both poor and favorable outcomes in disease progression. However, secretion of SPARC by cancer-associated fibroblasts (CAF) disrupts adhesion and increases the motility of TNBC in vitro, suggesting SPARC is favorable for disease progression [51]. Overexpression of NID-1 in TNBC in vivo models promotes lung metastasis and tumor growth, whereas nidogen knockdown decreases motility and invasion of claudin-low breast cancer cells, which are characterized by depletion of tight-junction proteins [52, 53]. The abundance of obesity-associated ECM accessory proteins HA and endotrophin is associated with poor disease outcomes in pan-subtype analyses of breast cancer patients, while expression of heparanase, which regulates ECM at the cell surface, is predictive of poor prognosis in HR+ breast cancer [54–56]. Conversely, expression of the accessory protein decorin (DCN) is elevated with obesity and has a positive prognostic value [57, 58]. These findings highlight the unique ECM landscape of breast tissue conditioned by obesity, including increased collagen composition and basement membrane expansion. The consequences of these pre-existing alterations depend on the ratio of favorable and unfavorable ECM components for breast cancer development and progression.

In breast cancer, obesity-induced ECM remodeling promotes a pro-tumorigenic environment. When proteomic data from decellularized MFP of mice fed a HFD were compared to proteomic data from mouse mammary tumors, Wishart et al. identified nine overexpressed proteins in both the tumor and obese MFP ECM. These proteins include collagen IV, collagen XII, FN1, laminin subunit alpha 5 (LAMA5), vitronectin (VTN), elastin (ELN), von Willebrand factor A domain 1 (VWA1), galectin-1 (LGALS1), and annexin A3 (ANXA3) [45]. Of these proteins, expression of collagen IV, collagen XII, FN, VTN, and ANAX3 are indicators of unfavorable breast cancer outcomes (Table 1). Collagen IV, a prominent basement membrane collagen, is associated with cell motility and poor chemotherapeutic outcomes [59, 60]. High collagen XII is a predictor of poor overall survival and progression-free survival, especially for patients with early-stage breast cancer [61]. FN1 is required for the assembly of collagen I fibers and has been shown to induce epithelial to mesenchymal transition of HR+ breast cancer cells [62]. ANXA3 promotes heterogeneity of breast cancer stem cells and is associated with chemoresistance; knockdown of ANXA3 prevents lung metastasis in vivo [63]. The impact of compositional changes to the ECM in the obese stroma is multi-faceted, trending towards an environment favorable for breast cancer progression.

**Table 1:** Summary of ECM modifications during obesity and breast cancer.

ECM properties	Obesity	Breast cancer	Prognosis	Associated with drug resistance	Source
Collagen alignment/linearity	↑	↑	Unfavorable	-	[115, 122, 123]
Stiffness/ECM deposition	↑	↑	Unfavorable	Yes	[5, 43, 44]
LOX expression	↑	↑	-	Yes	[80, 124]
Fibril-associated proteins					
Collagen XII	↑	↑	Unfavorable	-	[24, 61]
Von Willebrand Factor-1	↑	↑	-	Yes	[24, 125]
Tropoelastin	↑	↑	-	-	[45]
Structural proteins					
Collagen I	↑	-	Treatment- Dependent	Yes	[45, 48, 126]
Collagen III	↑	-	Favorable	-	[45, 49]
Collagen IV	↑	↑	Unfavorable	Yes	[45, 60, 127]
Collagen VI	↑	↑	Unfavorable	-	[45, 128–131]
Collagen VII	↑	-	Favorable	-	[45, 50]
Glycoproteins					
Fibronectin type 1	↑	↑	Unfavorable	Yes	[45, 132–136]
Laminin alpha- 2	↑	-	Unfavorable	-	[45, 137]
Laminin alpha-4	↑				[19, 20]
Laminin alpha-5	↑	↑	-	-	[45]
Laminin subunit gamma-1	↑	↑	-	-	[18, 138]
Vitronectin	↑	↑	Unfavorable	-	[45, 139]
Nidogen	↑	-	Unfavorable	-	[18, 52]
Fibrillin-1	↑	↑	-	-	[140]
Tenascin C	↑	↑	Unfavorable	Yes	[82, 141, 142]
Metalloproteinases					
MMP-2	↑	↑	Unfavorable	Yes	[143–147]
MMP-9	↑	↑	Unfavorable	Yes	[143, 145, 147, 148]
MMP-14	↑	↑	Unfavorable	Yes	[147, 149, 150]
Accessory proteins					
Galectin-1	↑	↑	-	Yes	[45, 151]
Annexin a3	↑	↑	Unfavorable	Yes	[45, 84]
Heparanase	↑	-	Unfavorable	Yes	[45, 56]
Hyaluronan	↑	-	Unfavorable	-	[45, 54]
Elastin	↓	↑	-	-	[152]
Endotrophin	↑	↑	Unfavorable	Yes	[55, 153, 154]
Secreted protein rich in cysteine	↑	↑	Unfavorable	-	[18, 155]
Heparin sulfate proteoglycan-2	↑	↓	Unfavorable	-	[18, 156]
Transforming growth factor beta	↑	↓	Unfavorable	Yes	[45, 157, 158]
Decorin	↑	↓	Favorable	Protective against	[57, 58, 159]

The ECM undergoes significant remodeling in both the obese and cancerous breast adipose tissue. The combination of these changes varies in prognostic and therapeutic impact, ranging from favorable to unfavorable for breast cancer outcome and for treatment resistance.

## Onco-architecture of the obese and cancerous ECM

### Fiber dynamics in the obese stroma and tumor

The alignment and orientation of fibrous proteins within the ECM are equally as important as composition to adipose tissue function and ECM-tumor interactions. These features are altered concurrently by obesity and tumor cells, depending on tumor proximity and stage. In non-cancerous tissue, obesity promotes a random deposition of collagens from ASCs, as defined by a decreased anisotropy coefficient, a measure of directional dependency. Collagen and fibronectin fibers deposited from obese ASCs are thicker than lean, as demonstrated by immunofluorescence and histological staining [3, 64]. Supporting this finding, Amens et al. found that collagen fibers derived from mouse pups with a maternal background of obesity are thicker than those from pups with mothers fed standard chow. HFD, regardless of a maternal background of obesity, enhances the thickness of collagen fibers. Further, collagens were described as curvier and more interconnected, suggesting a more random distribution of collagens in obese animals than in lean [6]. In a COL1A1 PyVT mouse model, high mammographic density increases the tumor initiation rate, indicating that changes to the fibrillar architecture by obesity, like collagen thickening, may provide an environment conducive to breast cancer development [65].

At the interface between the tumor and the stroma, there are local collagen patterns termed tumor-associated collagen signatures (TACSs) that represent collagen deposition and alignment from different stages of breast cancer. TACS1 and 2 include densely deposited collagen adjacent to the epithelium or straightened (“taut”) collagen deposits, respectively. TACS3 occurs as the tumor becomes invasive, characterized by aligned collagen fibers that orient parallel to invasive cancer cells [9]. TACSs demonstrate that the local alignment of collagen fibers at the tumor-stromal interface are associated with breast cancer cell metastasis and invasion [9].

Although aligned collagen is conducive to breast cancer progression, Amens et al. found that decellularized mammary fat pads derived from obese mice, which have an increased curvature and more random deposition, enhance the migration of MDA-MB-231 TNBC cells. This effect is attenuated when matrices are stretched to mimic aligned matrices found in lean mice, suggesting that obesity-induced curvature of collagens supports breast cancer progression. The group’s work represents earlier stages of breast cancer, where collagens are less conditioned by invasive breast cancer cells [6]. In 3D collagen matrices with a random alignment, there is an elevated number of MDA-MB-231 cell protrusions compared to aligned matrices; however, the formation of new protrusions into the collagen matrix limits TNBC cell migration [66]. In vivo depletion of Collagen III, which is upregulated in obese adipose, promotes the alignment of fibers deposited in the tumor stroma and a TACS-3 signature, suggesting the role of collagen III in random deposition [49]. In non-malignant mammary tumors, obesity increases the thickness of collagens. Interestingly, collagen thickening is associated with a larger collagen pore size, and both these parameters increase the invasion of 3D breast cancer organoids derived from a MMTV-PyMT mouse model [10]. While most studies indicate that obesity promotes collagen thickening and random deposition, there are contrasting views on how the structural features of collagen in the obese TME impact tumor progression. Furthermore, alignment and curvature are reported to stimulate cancer. To decipher how breast tumors respond to an altered fiber architecture, future studies must account for breast cancer subtypes, proximity to the tumor, and TACS-based classifications.

### Stiffness, mechanical alterations, and cancer-cell mechano-sensing

The ECM contains structural elements that allow cells to evaluate stiffness and regulate cell function through the use of force-sensing machinery [67]. Reorganization, degradation, or increased deposition of ECM can activate cell mechano-transduction within the tumor stroma, initiating changes in cell proliferation, viability, or adipose stem cell differentiation [68, 69]. Evaluation of the mechanical properties of whole or local adipose tissue provides insight into its ECM-induced macro- and microscopic properties [70, 71]. Mechanical testing can also monitor disease-related alterations to the ECM and assess healthy tissues, as well as changes to cellular processes. Conventional methods to evaluate tissue mechanics include tensile, indentation, rheometric, and compression testing. These tests determine tissue stiffness, by Young’s modulus (E) or viscoelastic, storage or loss moduli, properties, influenced by the ECM organization and composition [70].

Structural and compositional changes in the adipose tissue matrix, such as obesity-induced remodeling, significantly affect tightly balanced homeostatic processes. Obesity increases fibrosis and surrounding tissue rigidity through excessive deposition of collagen. For example, upregulation of collagen I was shown to elevate adipose tissue E, or stiffness [8]. ECM derived from mice fed a HFD is stiffer (higher E) than that from lean counterparts but has a lower ultimate tensile strength (UTS), the amount of stress tolerated before breaking [72]. Matrix stiffness increases in diabetic mice, and interestingly, positively correlates with the number of obesity-associated comorbidities [72, 73]. Obesity-induced stiffness can be reversed with surgical or caloric interventions [72]. Matrix stiffening is driven, in part, by the crosslinking of collagens associated with obesity. The crosslinking enzyme lysyl oxidase (LOX) is highly expressed in obese adipose tissue, leading to a higher proportion of nonreducible, crosslinked collagen and increased matrix stiffness [3]. In adipose tissue from C57BL/6 J mice, HFD-induced inhibition of TGFβ/AMPK promotes expression of LOX, which indicates enhanced crosslinking and stiffness within the tissue [3].

Obesity-driven ECM remodeling is well-documented in breast cancer progression, poor patient survival, and increased drug resistance. Recent reviews have focused on elucidating the obesity-breast cancer relationship, synergistic ECM changes [74] or stromal components [75], and even establishing drug targets to reduce ECM stiffness [76]. For example, obesity-associated ASCs, in ob/ob mice fed HFD, over-deposit unfolded and stiffer ECM, significantly increasing the average E in decellularized ECM (250 vs. 100 Pa) and interstitial ECM (21 vs. 12 kPa) compared to wild type. This significant change results in a tumorigenic phenotype that matches obese human breast tissue [5]. In analyses of cultured cancer cells with varied invasive potential on soft ( ~ 400 Pa) and stiff ( ~ 1200 Pa) collagen type I hydrogels, malignant cell lines (MDA-MB-231s and 4T1s) are more pliable, responsive, and mobile to positive changes in stiffness [8]. Independent of obesity, increases in matrix stiffness intensify cancer cell stemness [12] and/or YAP/TAZ pathway activation [77, 78], promoting breast cancer progression and drug resistance. Furthermore, increased LOX expression induces matrix crosslinking and stiffness, promoting tumor growth and metastasis in mice injected with HR+-derived organoids [79]. LOX expression and collagen crosslinking in in vivo TNBC xenograft models additionally hyperactivate integrin signaling and promote chemoresistance [80]. The synergism between obesity and breast cancer warrants both to be considered in future studies to maintain clinical relevance. Due to their linked actions, addressing obesity-associated morbidities will be paramount to the success of future BC clinical interventions and therapies.

## Interplay between obese-ECM, stromal cell types, and breast cancer

### Adipocytes

**Fig. 3:**
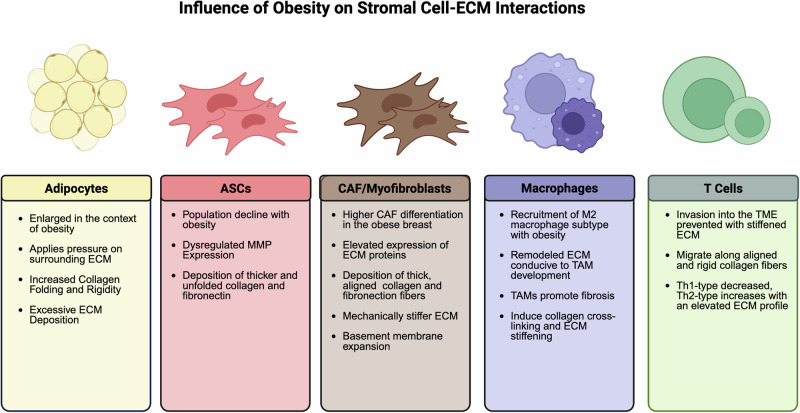
Influence of obesity on stromal cell-ECM interactions. Obesity-educated stromal cells aberrantly deposit ECM that further contributes to a pro-tumorigenic onco-architecture. Deposition of matrix by these cell types is dense in collagen and fibronectin, which are thicker, leading to a mechanically stiffened ECM. These collagens are either folded (adipocyte) or unfolded (ASC, CAF) depending on the cell type, highlighting that obesity has unique local impacts. Obese stromal cells further induce crosslinking of existing ECM and expansion of basement membrane components that exacerbate fibrosis, ECM rigidity, and promote breast cancer progression.

The compositional and physical properties of obesity-derived adipocytes dictate structural changes to the breast ECM (Fig. 3). Adipocyte hyperplasia requires an increase in ECM flexibility. Obese mice have significantly larger adipocytes than non-obese mice and stiffened and nonreducible collagen deposits surrounding obese adipocytes induce mechanical stress, metabolic dysregulation, and inflammation within the stroma [3, 16, 81]. As adipocytes increase in size, pressure on differentiated collagen rises, yielding folded and rigid collagen fibers [3]. The resulting ECM is net-like, which may further contribute to stiffening during HFD-induced obesity [81]. Formation of this net-like structure and a heightened stiffness are partly due to increased lipid droplet size within obese adipocytes [81]. Obesity is additionally associated with inflammation within the adipose tissue. For example, the proteoglycan tenascin C (TNC) initiates release of the pro-inflammatory cytokine tumor necrosis factor-alpha (TNF-α) from obese, visceral adipocytes via activation of toll-like receptors [82]. Gene signatures of obese murine adipocytes closely resemble pre-adipocytes or fibroblasts, characterized by excessive ECM gene expression and decreased lipolytic function [83]. Adipocytes isolated from mice fed HFD are deeply embedded within ECM, with a thicker layer of ECM surrounding and between adjacent adipocytes than in mice fed standard chow [16].

In co-culture with adipocytes from obese patients, breast cancer cell proliferation, migration, and invasion are significantly increased [74]. Obese adipocytes promote the proliferation of breast cancer cells in a TAZ/Resistin-dependent manner and enhance TNBC cell stemness [84]. Obesity induces the transformation of adipocytes to a more fibroblastic state, suggesting an enhanced capacity for ECM deposition that is favorable for disease progression [83]. While the implications of obesity-induced de-differentiation of adipocytes on breast cancer are unknown, adipocyte de-differentiation drives tumor progression and pro-tumor metabolic reprogramming [85].

### Adipose stromal cells

ASCs are multipotent cells that can differentiate into osteocytes, adipocytes, chondrocytes, and, in the context of breast cancer, cancer-associated fibroblasts (CAFs) [86, 87]. ASCs are one of the main matrix-producing cell types within the tumor and are conditioned by obesity status (Fig. 3) [87]. While adipose tissue expands with obesity, the ASC population declines, as evidenced in mice fed a long-term HFD. In these animals, MMP9 expression is markedly decreased, while MMP2 is increased. This alteration is not reversed by exercise [88, 89]. ASCs conditioned by obesity, collected adjacent to breast tumors, are proinflammatory, less stem-like, and have a depleted differentiation capacity compared to those from lean individuals [87]. ASCs derived from mammary fat pads of *ob/ob* mice deposit thicker and partially unfolded ECMs rich in collagen and fibronectin, contributing to matrix stiffening associated with obesity [5]. Expression of genes associated with ECM organization, assembly, and alignment is elevated in visceral ASCs isolated from obese individuals compared to those isolated from lean individuals. Additionally, these ASCs have higher levels of senescent marker expression, a phenomenon mediated by the obese matrix through TGFβ-driven mitochondrial dysfunction [64]. In individuals with obesity, similar mitochondrial dysfunction and senescence of ASCs contribute to increases in the basement membrane component LAMC [90]. ECM accessory proteins additionally impact ASCs in obese adipose tissue. On the surface of murine ASCs derived from WAT, full-length, glyconated DCN is cleaved by metalloproteinase-14 (MMP14) to form a non-glyconated product that disrupts normal collagen assembly. DCN cleavage by MMP14 leads to the accumulation of truncated Collagen VI and enlarged adipocytes, a phenotype that may be exacerbated by elevated expression of these proteins in obese adipose tissue [91].

Decellularized scaffolds derived from obese, murine ASCs promote the proliferation of TNBC cells by increasing matrix stiffness, mechanical sensing, and enhanced nuclear localization of YAP/TAZ [3]. Obese ASCs promote invasion of HR+ breast cancer cells through direct cell-cell contact, MMP-dependent ECM remodeling, and collagen contraction. Collagen densely packs around obese ASC-HR+ cancer cell coculture spheroids with the MMP inhibitor Batimastat. In the same treated cocultures, the collagen deposits were more fragmented than those in spheroids with lean ASCs, suggesting differences in MMP-dependent ECM remodeling with obesity [92]. Moreover, obese ASCs drive metastasis and promote estrogen independence of HR+ breast tumors. In HR+ wild-type cells and mutants with constitutive activation of the estrogen receptor, plasminogen activator inhibitor-1 (PAI1) is upregulated in the presence of obese ASCs. PAI1 inhibits urokinase plasminogen activator (uPA), a prominent regulator of extracellular matrix remodeling [93].

### Cancer-associated fibroblasts/myofibroblasts

Fibroblasts are one of the main matrix-producing cell types in the tumor stroma and are altered by obesity. Fibroblasts in the tumor stroma can transform into pro-tumor CAFs, which adopt myofibroblastic properties and promote ECM stiffening, inflammation, and tumor progression. CAFs originate from resident fibroblasts, ASCs, pericytes, and tumor/endothelial cells undergoing EMT. ASC-derived CAFs do not have the multipotent properties of their progenitors and are, therefore, a distinct cell type [94]. While there are several subtypes of CAFs, there is no consensus for categorization, with most studies defining CAF subtypes as either myofibroblastic (matrix-producing) or inflammatory (immune-modulating) [95]. Histological analysis of tumor-adjacent adipose tissue in TNBC indicates that levels of normal fibroblasts are comparable between obese and lean donors, but vimentin-positive CAF-like cells are more abundant in obese tissues, suggesting the propensity of obese fibroblasts for CAF development [96].

In studies conducted with ASCs isolated from breast tumors, obese, tumor-proximal ASCs express genes associated with myofibroblastic CAF differentiation to a higher degree than lean proximal ASCs [87]. Moreover, obese, abdominally derived ASCs express a greater incidence of CAF markers than lean when exposed to HR+ breast cancer cells [97]. Stromal fibroblasts from mice with obesity contribute to basement membrane expansion and abundant ECM deposition, increasing SPARC, ELN, COL3A1, and COL6A1 gene expression [98]. ASCs isolated from the mammary fat pads of *ob/ob* mice have enhanced expression of myofibroblast marker alpha-smooth muscle actin (αSMA) and deposit collagen and fibronectin-rich ECMs with thicker fibers [5]. Additionally, myofibroblasts from mice with obesity deposit excess aligned collagens and fibronectin, which render the surrounding ECM mechanically stiffer than in lean mice [99]. The resulting stiffened ECM not only exacerbates tissue inflammation but also promotes tumor malignancy and breast cancer progression [99]. One mechanism of myofibroblastic CAF differentiation from human subcutaneous ASCs is through TGFβ paracrine signaling with breast cancer cells [100]. RNA-seq data from malignant and normal tissue reveal that the ECM signature of CAFs overlaps with that from malignant tissue, and this expression profile is activated with TGFβ signaling [101]. These data suggest that the obese TME is more predisposed to CAF development, which fuels pro-tumorigenic ECM remodeling (Fig. 1).

### Tumor-associated macrophages

Macrophages are heterogeneous, with a high degree of interchangeability [102]. There are two classifications of macrophages, M1 and M2. The M1 phenotype promotes classic activation and is antitumor and pro-inflammatory [103]. The M2 phenotype promotes alternative activation and is protumor and anti-inflammatory [104]. The M2 phenotype is the predominant subtype recruited and generated in obese conditions (Fig. 1) [105]. M2 macrophages are enriched in obese breast tissue and are more likely to form tumor-associated macrophages (TAMs). Upregulation of M2 marker resistin-like molecule alpha (FIZZ1) and chemokine ligand 6 (CCL6) with obesity suggests M2 recruitment, immunosuppression, and an environment predisposed to TAM development in the mouse MFP [98].

Studies show that TAMs promote fibrotic ECM remodeling within the TME, and recent literature suggests that profibrotic ECM remodeling itself can encourage a TAM-like macrophage phenotype, fueling a positive feedback loop [99]. In obese adipose tissue, upregulation of the chemokine CCL2 induces chronic macrophage-driven inflammation, and similarly, CCL2 overexpression in the TME stimulates infiltration of TAMs and excessive collagen deposition [106]. Comparably, the depletion of macrophages using clodronate-containing liposomes in mice with EO771 (HR + ) tumors alters collagen fibrillar microstructure [107]. TAMs within tumors drive the crosslinking of collagen fibers and ECM stiffening through the secretion of TGFβ, particularly in TNBC, which contains a higher proportion of macrophage infiltration than other subtypes [17].

### T-helper cells

Breast cancer prognosis improves with the infiltration of Th1-type T-helper cells within the TME. In more aggressive breast cancer subtypes, such as TNBC, therapeutic agents such as PD-1 inhibitors restore the action of these cells and prevent the immune evasion of cancer cells [108]. Mechanical changes to ECM stiffness in obese individuals could exacerbate immune evasion as matrix stiffness prevents the migration of T cells and their invasion into the TME [108–110]. In MMTV-PyMT tumors treated with the LOX inhibitor BDPN, which decreases the percentage of matrix with a stiffness over 40 kPa, there is a higher degree of T cell infiltration and T cell migration speed than in untreated tumors [111]. T cells preferentially migrate along aligned and rigid collagen fibers, suggesting that the random deposition of collagens within the TME of obese patients could prevent their accessibility to tumors [110]. The obese adipose ECM is densely packed with enhanced expression of ECM proteins. In patients with an elevated ECM profile, based on genomic data from The Cancer Genome Atlas (TCGA) and Gene Expression Omnibus (GEO), there are fewer tumor-suppressive Th1 cells and more pro-inflammatory Th2-type T-helper cells (Th2), with negative impacts on prognosis [108]. The interplay between ECM mechanics and T cell infiltration in breast cancer warrants further investigation into the role of obesity. Future studies should also highlight the spatial heterogeneity of the obese ECM, as T cell migration is impacted by fiber alignment [110].

## Dual effects of aging and obesity on ECM and breast cancer

Sufficient collagen levels in the ECM are necessary for tissue health, and the proportion of collagen in the stroma decreases with age [112, 113]. Collagen is modified through MMP-targeted degradation, an effect with negative impacts for fiber stability [114]. Collagen can be modified by mineralization and advanced glycation end-products (AGEs), which drive the formation of cross-links. Collagen fibers altered in this way are associated with decreases in collagen lysis and, therefore, a reduction in MMP-mediated tissue remodeling [114]. Bahcecioglu et al. show that with aging, collagen fibers in breast tissue become thinner and curvier, and fatty components in the ECM increase, enhancing ECM stiffness [115]. Accumulation of fragmented collagen and protein aggregates during aging can additionally deteriorate ECM dynamics and promote stiffness [116, 117]. Concurrently, obesity increases the deposition of collagen and fibronectin fibers, leading to a denser and stiffer ECM [5]. Aging additionally alters the composition and organization of the ECM, supporting the replacement of type V collagen fibers in the ECM with type I collagen fibers and fibrillin and decreasing local compact networks and structural ECM proteins [115].

In breast cancer, an aged matrix promotes mammary epithelial cell migration and invasion [115]. Bahcecioglu et al. further demonstrate that, in both normal KTB21 and cancerous MDA-MB-231 human mammary epithelial cells, aged breast ECM induces an invasive and cancer-like phenotype through upregulation of LOX. LOX is heightened in normal and tumor breast tissue in obese patients by activation of hypoxia-inducible factor 1a (HIF-1a) which interacts with the hypoxia response element located in the LOX promoter region, suggesting that combined obesity and aging promote crosslinking [118, 119] In addition, the aged ECM is associated with higher levels of proinflammatory cytokine release, which encourages cancer cell invasiveness and cell migration [115]. With increasing age, mesenchymal stem cells (MSCs) become senescent and less motile while maintaining ECM remodeling activity, prompting gene dysregulation and upregulation in LOX and tissue inhibitors of metalloproteinases (TIMPs) [120]. The increased activity of LOX and TIMPs, along with dysregulation of other ECM proteins, creates an abnormal collagen architecture and ECM stiffening [120]. MSC-induced matrix remodeling increases the proliferation and migration of breast cancer cells, suggesting a more aggressive tumor biology [120]. This illustrates that obesity and aging together promote ECM remodeling and potentiate an aggressive breast tumor phenotype.

## Obesity-induced ECM changes and drug response

ECM remodeling and ECM-cell interactions are factors contributing to the resistance of breast cancer to chemotherapeutic agents. In particular, increased ECM stiffness is associated with decreased efficacy of the anthracycline, doxorubicin [43, 44]. MDA-MB-231 cells encapsulated in a stiff ECM (9 kPa) are more resistant to doxorubicin treatment than those in a soft ECM (0.4 kPa), evidenced by lower proportions of dead cells, higher expression of the anti-apoptotic protein BCL-2 and proliferation marker Ki67, and a higher IC_50_ value [43]. Nuclear EGFR is significantly upregulated in TNBC cells with stiffness-induced doxorubicin resistance. Sensitivity to treatment is, interestingly, rescued when these cells are treated with an EGFR inhibitor [43]. In accord with these findings, Joyce et al. demonstrate that the LD_50_ value of doxorubicin more than tripled for MDA-MB-231 cells cultured in 200 kPa hydrogels as opposed to a 2000kPa hydrogel. Based on LD_50_ evaluation, cells are more sensitive to doxorubicin at 24 and 120 hours of treatment than at 2 hours, indicating attenuation of drug resistance as cells acclimate to ECM stiffness over time [78]. Further, the association between ECM stiffening and doxorubicin resistance is partly due to enhanced nuclear localization of YAP, decreased levels of E-Cadherin, and heightened EMT [78].

The composition of the ECM may account for differential drug response in breast cancer. Gurung et al. cultured HER2+ and TNBC cells with various ECM components to evaluate the impact on sensitivity to lapatinib, a tyrosine kinase inhibitor. HER2+ cells cultured with ECM protein laminin I exhibit morphological changes, increased migratory potential, and greater viability, an effect not reversed with Lapatinib treatment. MDA-MB-231 cells show no changes to proliferation or paclitaxel response with laminin I co-culture. These results suggest an ECM and subtype-specific impact on viability. Laminin I additionally improves recovery and replicative viability of HER2+ cells. In the presence of Lapatinib, cells transferred from laminin I-coated plates to other laminin I-coated plates regained viability to a higher degree than cells transferred from laminin I-coated plates to uncoated plates. These findings implicate the role of ECM in disease relapse following chemotherapy treatment [121].

Beyond resistance, newly developed diabetes and weight loss drugs may reduce the adverse effects of obesity-associated ECM remodeling. One study unveiled the immunohistochemical impact of Semaglutide, a GLP-1 agonist, on ECM remodeling in pancreatic islet cells of obese mice. They found decreased collagen type I, the alpha 3 chain of collagen type VI, LOX, and the collagen proteinases MMP-2 and MMP-9 in obese mice treated with Semaglutide compared to controls [122]. These findings suggest that Semaglutide has the potential to reverse pro-tumorigenic ECM remodeling associated with obesity, although more research needs to be conducted to determine if changes to ECM are a drug-specific effect or a result of weight loss. Moreover, coupled with the associations in this review linking collagen, LOX, and MMP upregulation with breast cancer, these results indicate the potential for GLP-1 agonist incorporation into combinatorial regimens as a therapeutic strategy for breast cancer in obese patients.

## Concluding remarks and future directions

This review highlights obesity as an important consideration for clinically relevant models and treatments of breast cancer, as it has complex implications for the disease. Obesity drives both direct modifications to tumor ECM and indirect stromal changes, which culminate in a stiffened, random, and highly crosslinked environment favorable for breast cancer. Stiffened ECM in response to obesity induces cell differentiation, malignancy, and immunosuppression, contributing to a positive feedback loop of aberrant ECM deposition and cell recruitment. These are exacerbated by age. While there are promising strategies to investigate the dual effects of obesity and breast cancer on ECM, further preclinical model development is needed to recapitulate the full complexity of the obese TME. Additionally, the effects of obesity treatments, namely semaglutide-based weight loss medications, on ECM remodeling and breast cancer treatment have not been explored. In focusing on the combined effects of obesity and breast cancer, this review discusses a limited portion of the ECM and accessory proteins involved in both. Further studies are needed to capture the full scope of ECM remodeling within the obese and cancerous breast. ECM within the breast adipose is dynamic, varying in patient-to-patient and obesity status. These pre-existing ECM alterations provide cues for breast cancer cell development, spread, and treatment response, prompting differential response with varied demographics. Therefore, for informed patient care, ECM and obesity must be highlighted within studies focused on breast cancer.
